# A decision aid to rule out pneumonia and reduce unnecessary prescriptions of antibiotics in primary care patients with cough and fever

**DOI:** 10.1186/1741-7015-9-56

**Published:** 2011-05-13

**Authors:** Johann Steurer, Ulrike Held, Anne Spaar, Birke Bausch, Marco Zoller, Roger Hunziker, Lucas M Bachmann

**Affiliations:** 1 Horten Center for Patient-Oriented Research and Knowledge Transfer, University of Zurich, Zurich, Switzerland; 2 Institute of General Practice and Health Services Research, University of Zurich, Zurich, Switzerland; 3 Division of Diagnostic and Interventional Radiology, University Hospital of Zurich, CH-8091 Zurich, Switzerland

## Abstract

**Background:**

Physicians fear missing cases of pneumonia and treat many patients with signs of respiratory infection unnecessarily with antibiotics. This is an avoidable cause for the increasing worldwide problem of antibiotic resistance. We developed a user-friendly decision aid to rule out pneumonia and thus reduce the rate of needless prescriptions of antibiotics.

**Methods:**

This was a prospective cohort study in which we enrolled patients older than 18 years with a new or worsened cough and fever without serious co-morbidities. Physicians recorded results of a standardized medical history and physical examination. C-reactive protein was measured and chest radiographs were obtained. We used Classification and Regression Trees to derive the decision tool.

**Results:**

A total of 621 consenting eligible patients were studied, 598 were attending a primary care facility, were 48 years on average and 50% were male. Radiographic signs for pneumonia were present in 127 (20.5%) of patients. Antibiotics were prescribed to 234 (48.3%) of patients without pneumonia. In patients with C-reactive protein values below 10 μg/ml or patients presenting with C-reactive protein between 11 and 50 μg/ml, but without dyspnoea and daily fever, pneumonia can be ruled out. By applying this rule in clinical practice antibiotic prescription could be reduced by 9.1% (95% confidence interval (CI): 6.4 to 11.8).

**Conclusions:**

Following validation and confirmation in new patient samples, this tool could help rule out pneumonia and be used to reduce unnecessary antibiotic prescriptions in patients presenting with cough and fever in primary care. The algorithm might be especially useful in those instances where taking a medical history and physical examination alone are inconclusive for ruling out pneumonia

## Background

Respiratory tract infections are the most frequent reasons for unnecessary antibiotic prescriptions and inappropriate treatment with antibiotics is one of the avoidable causes for the world-wide increasing problem of antibiotic resistance [1-3]. Guidelines [4-6] and patient information leaflets [7] emphasize this serious problem by recommending antibiotics only for patients with bacterial pneumonia and not for patients with acute bronchitis or mild exacerbations of chronic bronchitis [8,9]. The reasons for the still high rate of inappropriate prescriptions in patients with cough and fever are manifold [1]. An important and comprehensible one is that physicians do not want to miss the diagnosis of pneumonia. The problem in daily practice is that no single symptom or clinical sign is pathognomonic for the presence or absence of pneumonia, and differentiation between pneumonia and non-pneumonia based on single clinical signs is not possible.

While a normal chest x-ray, the reference test applied in clinical practice, rules out pneumonia with a high degree of certainty [10], recommendations discourage performing an x-ray in every patient presenting with a cough and increased body temperature. Costs are unjustifiably high and patients would be exposed to radiation unnecessarily [11]. An easy applicable diagnostic aid to support primary care physicians in ruling out pneumonia and to identify patients for whom treatment with antibiotics is not necessary would be helpful but is unavailable.

The aim of this study was the development of a diagnostic aid, based on clinical signs and the measurement of C-reactive protein, to support physicians to safely rule out pneumonia in patients with cough and fever and to help in reducing unnecessary prescriptions of antibiotics.

## Methods

The ethics committees of the cantons of Zurich, St. Gallen and Thurgau approved the protocol and we obtained informed consent from all participants.

General practitioners and directors of clinics in Internal Medicine in the eastern part of Switzerland were invited to participate in this study. They received a letter explaining the aim of the study. Physicians and directors, which confirmed participation, were provided with detailed information about the study, including the questionnaire to fill in information on patients' histories, findings on physical examination, lab results and X-ray findings. In addition, they obtained patient information leaflets and forms for written informed consent.

Patients aged 18 years and older with new or worsened (at least 24 hours) coughs and subjective or measured increased body temperatures were eligible to enter the study.

We excluded patients with known chronic lung diseases (except chronic bronchitis), patients who developed cough and fever during their hospital stay, patients with a positive HIV status or who were taking oral steroids within the last month, patients on chemotherapy, patients after organ transplantation, pregnant women, and patients with a mental disorder or those incapable of reading the information leaflet and/or giving informed consent.

After getting informed consent, physicians performed and recorded a standardized medical history and physical examination. In addition, venous blood samples for C-reactive protein were drawn from all patients and blood was analyzed using standard procedures. Chest radiographs, lateral and postero-anterior views, were obtained from every patient after clinical examination irrespective of whether the treating physician would have ordered them outside this study. Physicians were asked to rate infiltrates or other pulmonary abnormalities.

Physicians were free to order further tests; participating in the study had no influence at all on any treatment and care of patients. Moreover, the result of the decision rule was not available when treating patients.

The completed questionnaires and the radiographs were sent to the study centre at the University Hospital in Zurich. All radiographs were re-assessed by a senior staff radiologist at the University Hospital. The radiologist was blinded to the clinical information and the result of the assessment by the general physician. In accordance with the BTS Guidelines for the Management of Community Acquired Pneumonia in Adults we defined pneumonia as a set of symptoms and signs consistent with an acute lower respiratory tract infection associated with radiographic shadowing for which there is no other explanation (for example, no pulmonary oedema or infarction) [10,12].

The questionnaire, including all the relevant information to differentiate between pneumonia and acute bronchitis, was developed in an iterative process by clinical experts in the field [13]. The questionnaire, consisting of 25 items, asked for information about age, gender, symptoms (for example, duration of cough, sputum production, and self-reported fever), results of physical examination (for example, respiratory rate, abnormal breath sounds), the risk indicators (for example, smoking history, indications for upper respiratory tract infection), C-reactive protein level and findings in chest x-ray. (Items included in the questionnaire are shown in Table 1 together with summary statistics for patients with and without pneumonia, separately.)

**Table 1:** Comparison of patients with versus without pneumonia

Variate*	All patients (n = 621)	Patients without pneumonia (n = 494)	Patients with pneumonia (n = 127)
Age	46.7 (SD 16.3)	46.6 (SD 16.1)	46.8 (SD 17.2)
Gender (male)	308 (50%)	247 (50%)	61 (48%)
New onset/worsened cough's duration (days)	7.0 (SD 9.6)	6.7 (SD 6.4)	8.4 (SD 17)
Chronic cough	43 (7%)	30 (6%)	13 (10%)
Daily fever	350 (56%)	266 (54%)	84 (66%)
Maximum temperature (°C)	38.5 (SD 1.0)	38.4 (SD 1.0)	38.8 (SD 1.0)
Dyspnea	223 (36%)	165 (33%)	58 (46%)
Dyspnea at effort only	117 (19%)	88 (18%)	29 (23%)
Wheezing	109 (18%)	85 (17%)	24 (19%)
Pain on inspiration	179 (29%)	133 (27%)	46 (36%)
Rigors	205 (23%)	150 (30%)	55 (43%)
Muco-purulent sputum	302 (49%)	240 (49%)	62 (49%)
Bloody sputum	42 (7%)	27 (5%)	15 (12%)
Cold/Influenza signs	301 (48%)	252 (51%)	49 (39%)
Non-Smoker	440 (71%)	349 (71%)	91 (72%)
Smoking (cigs./day)	16.4 (SD 9.1)	16.1 (SD 9.3)	17.6 (SD 8.7)
Pack years	12.7 (SD 18.2)	8.7 (SD 12.2)	25 (SD 26.7)
History of pneumonia	20 (3%)	17 (3%)	3 (2%)
Current temperature (°C)	37.4 (SD 1.0)	37.3 (SD 1.0)	37.7 (SD 1.0)
Signs of upper respiratory infection	234 (38%)	201 (46%)	33 (26%)
Respiratory rate (#/minute)	17 (SD 6)	16.6 (SD 5.7)	18.6 (SD 5.9)
Prolonged expiration	64 (10%)	48 (10%)	16 (13%)
Percussion: dullness (a)	38 (6%)	13 (3%)	25 (20%)
Auscultation: friction rub (b)	18 (3%)	7 (1%)	11 (9%)
Auscultation: diminished inspiratory sound (c)	74 (12%)	43 (9%)	31 (24%)
Auscultation: bronchial breath sound (d)	52 (8%)	28 (6%)	24 (19%)
Auscultation: rales and/or wheezing (e)	192 (31%)	144 (29%)	48 (38%)
Abnormalities in a to e, if 2+ in single locus	140 (23%)	84 (17%)	56 (44%)
Abnormalities in a to e, if 2+ in different loci	18 (3%)	10 (2%)	8 (6%)
C-reactive protein (CRP)			
CRP 0 to 10	108 (17%)	108 (22%)	0 (0%)
CRP 11 to 50	265 (43%)	240 (49%)	25 (20%)
CRP 51 to 100	106 (17%)	78 (16%)	28 (22%)

CRP >100	134 (22%)	61 (12%)	73 (57%)

*Means and standard deviation; number of patients and percentages

### Statistical analysis

In a first approach, validating a previously published expert-based diagnostic algorithm [13], we fitted a multiple logistic regression model to the binary outcome variable pneumonia (yes/no). Independent variables were the original set of clinical-diagnostic indicators (25 variables) [13]. Additionally, we included C-reactive protein. Due to the fact that a small number of missing values occurred in some of the variables, and since we considered these values to be missing at random, we used a multiple imputation method based on chain equations [14]. We imputed five datasets, and we estimated the coefficients and their confidence intervals in the multiple models based on a pooled fit over all five of the imputed datasets. To quantify the discriminative ability of the multiple models, we calculated a pooled estimate of the area under the receiver operating characteristic curve (AUC) for the five imputed datasets along with the corresponding confidence interval.

We expected that our multiple model fits the data too well, in the sense that any "unusual random feature in the original data will be reflected in the predictions", but will not be replicated in a new set of observations [15]. Therefore, we calculated the shrinkage factor applying a leave-one-out cross validation in each of the five imputed datasets. In order to obtain a conservative estimate of the shrinkage factor, we kept the shrinkage factor with the largest regression slope and deduced shrunken regression coefficients for the final multiple model (0.85 (corresponding intercept was 0.03).

Second, we used Classification and Regression Trees (CART) in order to determine an easy-to use rule out criterion for pneumonia [16]. In contrast to the multiple models consisting of the complete set of 25 independent variables, we aimed to find an applicable decision tree in a GP setting. For that reason, we pre-selected six variables on the basis of ease of availability and reliability of the information from the original set of indicators, including chronic cough, daily fever, dyspnoea, respiratory rate, pleural friction rub, and C-reactive protein. When developing the first exploratory tree using all 25 clinical-diagnostic indicators, we assigned a higher cost to misclassifying a pneumonia-case than a non-case. We later reduced the complexity of the tree by pruning according to a complexity measure to get a decision tree. In order to gain some idea about the tree's validity, it was developed on one dataset obtained from multiple imputations and applied to the remaining four imputed datasets.

Finally, we assessed the number of patients classified as non-cases and the percentage of antibiotic prescriptions that, in retrospect, could have been avoided in this group. This means the diagnostic tool was applied post treatment to assess whether or not these patients actually needed antibiotic intervention. This was compared to whether or not the patients had actually received antibiotics. We tested whether the difference of the two sample proportions *p*_0_, *p*_1 _of antibiotic prescriptions with and without the CART decision rule was different from zero. For that we used the statistic , under the null-hypothesis that the true unknown proportions *π*_1 _= *π*_0 _= *π *[[17], p. 152].

## Results

A total of 245 physicians were invited to participate in the study; 120 confirmed their participation and 86 physicians enrolled 642 patients between November 2006 and December 2009. We asked them to include all eligible patients consecutively.

Twenty-one patients had missing information about pneumonia status and were not included in the final analysis. The mean age was 46.8 years (standard deviation (SD) 16.3), and 50% were male. A total of 598 patients attended a primary care physician and 23 (3.7%) an emergency department in a hospital. The mean duration of cough until consultation was seven (SD 9.6) days, in 548 (93%) cough was a symptom of recent onset and 43 (7%) patients had chronic cough indicating chronic bronchitis. All patients had subjective feelings of increased body temperature, at the time of clinical examination the mean body temperature was 37.4°C (SD 1.0). Body temperature at time of consultation was ≥38.5°C in 87 patients (14%). Detailed information about symptoms and signs are shown in Table 1.

Radiographic signs for pneumonia were present in 127 (20.5%) of patients. Physicians prescribed antibiotics to 355 out of 609 patients (58%) (Information on antibiotic prescription was missing for 12 participants). All but four patients with pneumonia and 234 (38%) of patients without radiological signs of pneumonia received a prescription for antibiotics. (Information on antibiotics prescription was missing in two cases with confirmed pneumonia)

The results of the regression model can be found in Table 2 and the final (shrunken) function is available in Additional file 1. The area under the receiver operating characteristic curve was 0.90 (95% CI: 0.87 to 0.93).

**Table 2:** Pooled fit of the regression model based on 5 imputations*.

Variable	OR	Lower 95% CI	Upper 95% CI	*P*
Intercept	0.000	0.000	45.159	0.142
Age	0.969	0.948	0.991	0.008
New onset/worsened cough's duration	1.030	0.996	1.065	0.084
Chronic cough	3.088	0.854	11.162	0.085
Daily fever	1.636	0.843	3.175	0.145
Maximum temperature (°C)	1.215	0.873	1.691	0.248
Dyspnea	1.270	0.535	3.017	0.586
Dyspnea at effort only	1.318	0.503	3.451	0.572
Wheezing	0.483	0.209	1.116	0.088
Pain on inspiration	0.767	0.385	1.529	0.448
Rigors	1.110	0.567	2.173	0.756
Muco-purulent sputum	0.626	0.344	1.138	0.124
Bloody sputum	3.071	1.150	8.203	0.025
Cold/influenza signs	0.644	0.326	1.271	0.200
Smoking	0.979	0.945	1.014	0.241
History of pneumonia	0.171	0.015	1.923	0.145
Current temperature (°C)	1.025	0.746	1.409	0.876
Signs of upper respiratory infection	0.622	0.304	1.272	0.188
Respiratory rate (#/minute)	1.041	0.988	1.097	0.128
Prolonged expiration	1.066	0.390	2.914	0.901
Percussion dullness (a)	2.214	0.761	6.440	0.144
Auscultation friction rub (b)	10.343	1.843	58.052	0.014
Auscultation: diminished inspiratory sound (c)	1.869	0.831	4.205	0.130
Auscultation: bronchial breath sound (d)	1.118	0.517	2.416	0.774
Auscultation: rales and/or wheezing (e)	0.886	0.403	1.950	0.763
Abnormalities in a to e, if 2+ in single locus	3.633	1.414	9.334	0.009
Abnormalities in a to e, if 2+ in different loci	4.472	0.337	59.294	0.223

CRP	1.017	1.012	1.022	0.000

Original dataset included 621 patients with data on pneumonia.

*Original dataset included 621 patients with data on pneumonia.

**Figure 1:**
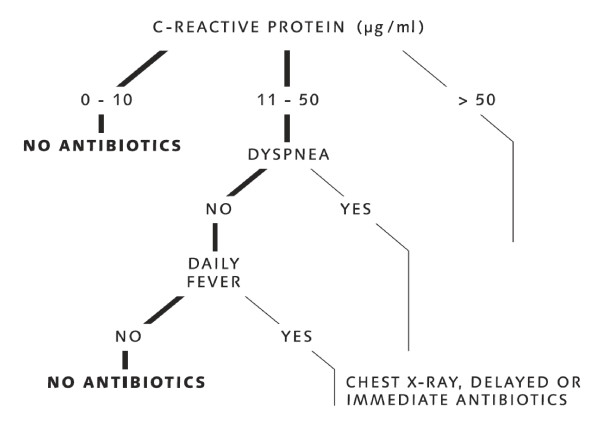
**Decision tree to rule out pneumonia**. C-reactive protein measured in μg/ml.

Figure 1 shows the classification tree. According to this tree, patients with C-reactive protein of below 10 μg/ml or patients presenting with C-reactive protein between 11 and 50 μg/ml, but without dyspnea and without daily fever can be classified as non-pneumonia cases and a prescription of antibiotics is not necessary. The complexity parameter of the tree was 0.04. The number and classification of patients within the tree is available in Additional file 2.

When we compared the proportion of antibiotic prescriptions with and without the classification rule, we found an overall potential reduction of antibiotic prescriptions of 9.1 percentage points (95% CI: 6.4 to 11.8; *P *< 0.001), and thus a significant reduction.

### Sensitivity analyses

In order to assess the robustness of our decision rule, we applied the tree to four datasets containing imputed values for missing data. In three of the four datasets, no single pneumonia case was misclassified when following the rule described above. In one of the datasets, we misclassified one single patient with pneumonia, because the corresponding C-reactive protein value was imputed with a value of <10 μg/ml.

We also assessed whether the enrolment site had an influence on our results. Repeating the analysis excluding 23 patients who had entered the study via the emergency department in a hospital had no influence on the reported findings.

## Discussion

We developed a diagnostic instrument (a prediction model) that allows calculating the probability of pneumonia in patients with new onset, or worsening, of a chronic cough and increased body temperature. We also derived a highly sensitive but simple clinical decision aid to support physicians in ruling-out pneumonia with a high degree of certainty and in consequence to reduce unnecessary prescriptions of antibiotics. When the C-reactive protein level is below 10 μg/ml or patients with C-reactive protein levels between 11 and 50 μg/ml do not complain about dyspnoea and daily fever since the onset of a cough, pneumonia can be ruled out with a high degree of certainty and unnecessary prescription of antibiotics could significantly be reduced.

### Our findings in the context of existing evidence

Our searches identified six prediction rules published in five papers to calculate the probability of pneumonia [18-22]. In three studies [19,21,22] only patients attending an emergency room and, in two of them, only patients for which the physician ordered a chest x-ray were included. In the study published by Melby, [20] patients with normal blood sedimentation rate and/or C-reactive protein levels were not included. These selections of patients may limit the application of the published prediction rules in a primary care population. Validation of these prediction rules in a small sample of primary care patients showed only a moderate ability to discriminate between patients with and without pneumonia [23]. In the prediction rule derived from primary care patients published by Hopstaken, dry cough, diarrhoea and fever ≥38.5°C were indicators remaining in the final multivariate model and C-reactive protein was the strongest indicator as in our study [18].

### Strength and limitations

The strength of our study is that over 90% of patients were included by general practitioners and the reference test for pneumonia was performed in all patients, minimizing verification bias. However, the prevalence of pneumonia in our sample is higher than in most other studies. The prevalence of pneumonia in patients with cough and fever ranges between 3 and 20% [24,25]. In a study also performed in the primary care setting, the prevalence was 13% [18]. The higher prevalence in our population has at least two reasons. One is the fact that the mean duration of cough until consulting a physician was seven days, indicating more severe or protracted respiratory tract infections. The second reason might be that physicians did not include eligible patients consecutively, but rather included patients with a longer duration of symptoms and probably higher suspicion of pneumonia. Therefore, the results of our study are more applicable to patients with a more severe or protracted course of infection of the lower respiratory tract and not for all patients with new onset of cough and increased body temperature.

A limitation of our study is that the results have not been validated in a new, similar set of patients in general practice. The internal validation techniques we used are inferior to external validations. Not gathering information about whether patients have diarrhoea is another weakness of our study. This symptom has been shown to be a relevant variable [18] in another prediction rule. Finally, we did not assess the diagnostic value of Procalcitonin, a relatively new marker for bacterial infections, although it might be a better test than the C-reactive protein to identify patients who should be treated with antibiotics [26,27]. We decided against Procalcitonin because the C-reactive protein is a widely used test which requires only a finger prick and the result is available within a few minutes. In addition, compared to Procalcitonin the cost for C-reactive protein measurement is much lower. Arguably, assessing Procalcitonin instead of C-reactive protein levels would have improved our instrument.

### Implications for practice

When physicians, after taking history and physical exam, are in doubt whether pneumonia is present and prescription of antibiotic treatment is indicated or not, measuring the C-reactive protein concentration and using the decision rule may be helpful in clinical practice. The rule helps in identifying a group of patients where pneumonia is very unlikely and chest X-rays and antibiotics are unnecessary. For the remaining patients in which pneumonia cannot be ruled out, physicians will order an X-ray and/or write a prescription for antibiotics and instruct them to start with antibiotics immediately or after some days if symptoms remain.

Unnecessary prescription of antibiotics contributes to the development of antibiotic resistance in the individual patient with consequences on the societal level. It is estimated that in the USA antibiotic resistance costs between $21 to $34 billion annually [28]. Use of this easy applicable decision aid by general practitioners will contribute to reducing the worldwide manifest problem of antibiotic resistance. Infections of the respiratory tract are one of the most frequent reasons for inappropriate antibiotic prescriptions worldwide and even a minor reduction in prescriptions of 10% could have major consequences for individual patients and on the societal level over time.

### Implications for research

In further studies the decision aid to rule out pneumonia should be validated in primary care patients with cough and fever. Also, the probability function should be validated in a new sample of patients. In further studies an optimal test-treatment strategy for patients in which pneumonia cannot be ruled out with the decision aid should be developed and validated by taking into account the adverse effects of inappropriate prescriptions of antibiotics and unnecessary chest X-rays.

## Conclusions

Following validation and confirmation in new patient samples, the decision aid to rule out pneumonia in patients with a new onset of cough or worsening of chronic cough and increased body temperature could lead to a reduction in unnecessary prescription of antibiotics. Applying this fast and frugal algorithm, physicians may reduce over-prescription of antibiotics and contribute to decelerate the growing problem of bacterial resistance.

## Abbreviations

AUC: area under the receiver operating characteristic curve; CART: Classification and Regression Trees; GP: General Practitioners; SD: Standard Deviation; 95% CI: 95% Confidence Interval

## Competing interests

The authors declare that they have no competing interests.

## Authors' contributions

All authors were involved in the conception and design, or analysis and interpretation of data. JS and LMB drafted the article and all authors revised it critically for important intellectual content and gave final approval of the version to be published. JS is the guarantor of this paper.

## Pre-publication history

The pre-publication history for this paper can be accessed here:

http://www.biomedcentral.com/1741-7015/9/56/prepub

## Supplementary Material

Additional file 1 **This file provides the complete model along with the shrunken coefficients**. Click here for file

Additional file 2 **This file shows the classification tree to rule out pneumonia**. Click here for file
