# Supplementary material for: A decision aid to rule out pneumonia and reduce unnecessary prescriptions of antibiotics in primary care patients with cough and fever
Source: BMC Med. 2011 May 13;9:56. doi: 10.1186/1741-7015-9-56 (PMC3118372; doi:10.1186/1741-7015-9-56)
Supplement: Additional file 1 — This file provides the complete model along with the shrunken coefficients. [file 1741-7015-9-56-S1.DOCX]

**Additional file 1** provides the complete model along with the shrunken coefficients. Individual probabilities can calculated with: P = 1/[1 + exp(–S)].

S= -9.584 - Age x 0.026 + New onset/worsened cough’s duration x 0.025 + Chronic cough x 0.958 + Daily fever x 0.418 + Maximum temperature (°C) x 0.165 + Dyspnea x 0.203 + Dyspnea at effort only x 0.235 - Wheezing x 0.618 - Pain on inspiration x 0.226 + Rigors x 0.089 - Muco-purulent sputum x 0.399 + Bloody sputum x 0.954 - Cold/influenza signs x 0.374 - Smoking x 0.018 - History of pneumonia x 1.501 + Current temperature (°C) x 0.021 - Signs of upper respiratory infection x 0.404 + Respiratory rate (#/min) x 0.034 + Prolonged expiration x 0.054+ Percussion dullness (a) x 0.675 + Auscultation friction rub (b) x 1.986 + Auscultation: diminished inspiratory sound (c) x 0.532 + Auscultation: bronchial breath sound (d) x 0.095 - Auscultation: rales and/or wheezing (e) x 0.103 + Abnormalities in a to e, if 2+ in single locus x 1.097 + Abnormalities in a to e, if 2+ in different loci x 1.273 + CRP x 0.014
