# Supplementary figures and images for: A decision aid to rule out pneumonia and reduce unnecessary prescriptions of antibiotics in primary care patients with cough and fever
Source: BMC Med. 2011 May 13;9:56. doi: 10.1186/1741-7015-9-56 (PMC3118372; doi:10.1186/1741-7015-9-56)

**Additional Figure** Classification tree to rule-out Pneumonia. CRP measured in μg/ml.


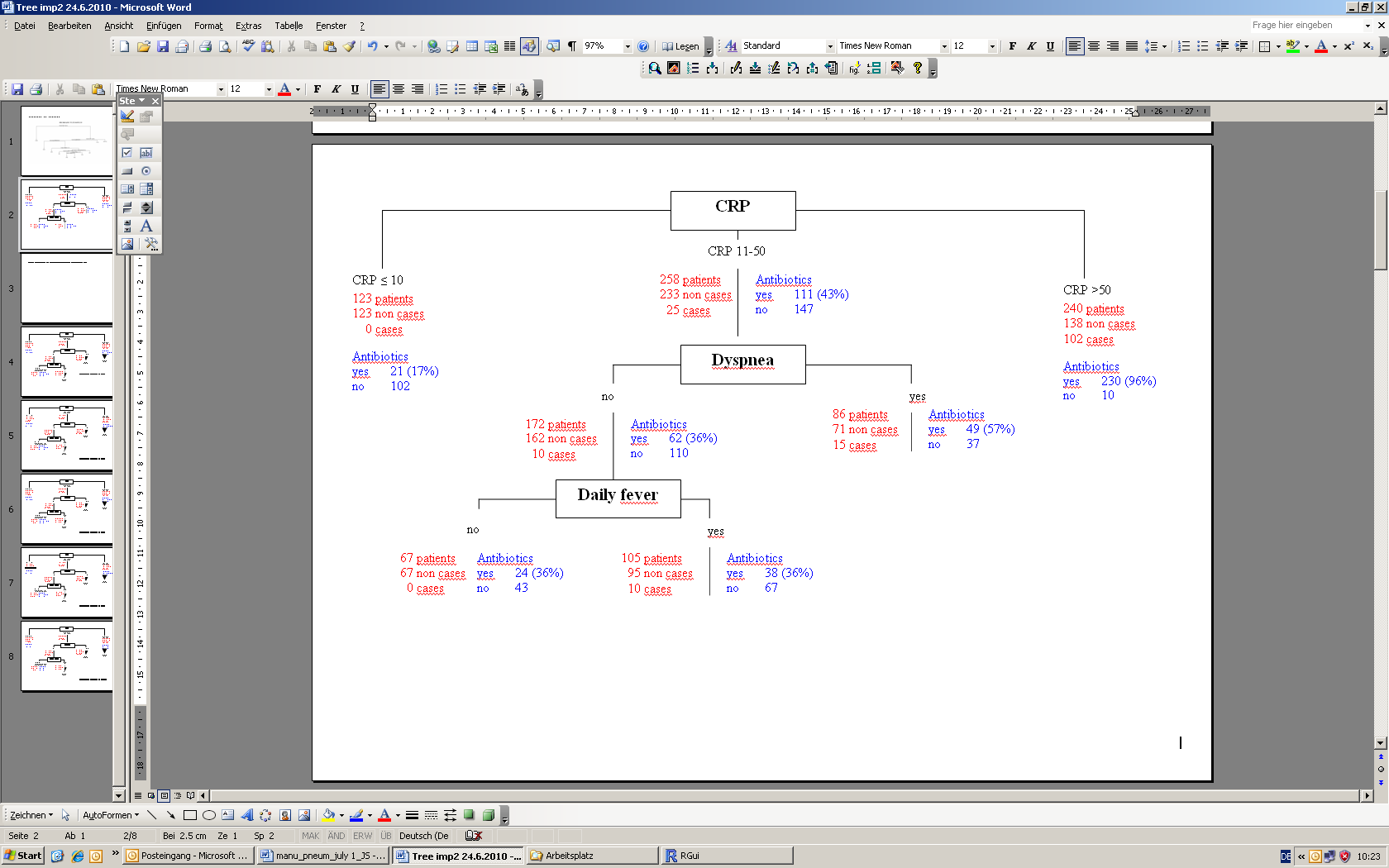

Supplement: Additional file 2 — This file shows the classification tree to rule out pneumonia. [file 1741-7015-9-56-S2.DOCX]
